# Discovery of anti-SARS-CoV-2 S2 protein antibody CV804 with broad-spectrum reactivity with various beta coronaviruses and analysis of its pharmacological properties *in vitro* and *in vivo*

**DOI:** 10.1371/journal.pone.0300297

**Published:** 2024-12-02

**Authors:** Yoji Tsugawa, Kentaro Furukawa, Tomoko Ise, Masahiro Takayama, Takeshi Ota, Takayuki Kuroda, Shinya Shano, Takashi Hashimoto, Haruyo Konishi, Takeshi Ishihara, Masaaki Sato, Haruhiko Kamada, Keita Fukao, Takao Shishido, Mai Yoshikawa, Tatsuya Takahashi, Satoshi Nagata

**Affiliations:** 1 Shionogi Pharmaceutical Research Center, Osaka, Japan; 2 Laboratory of Antibody Design, Center for Drug Design Research, National Institutes of Biomedical Innovation, Health and Nutrition, Osaka, Japan; 3 Shionogi TechnoAdvance Research, Co., Ltd., Osaka, Japan; Sungkyunkwan University - Suwon Campus: Sungkyunkwan University - Natural Sciences Campus, REPUBLIC OF KOREA

## Abstract

The SARS-CoV-2 pandemic alerted the potential for significant harm due to future cross-species transmission of various animal coronaviruses to human. There is a significant need of antibody-based drugs to treat patients infected with previously unseen coronaviruses. In this study, we generated CV804, an antibody that binds to the S2 domain of SARS-CoV-2 spike protein, which is highly conserved across the coronavirus family and less susceptible to mutations. CV804 demonstrated broad cross-reactivities not only disease-associated human beta coronaviruses including SARS-CoV, MERS-CoV, HCoV-OC43, HCoV-HKU1 and with existing mutant strains of SARS-CoV-2 and but also with 20 representative animal-origin coronaviruses. CV804 exhibits strong antibody-dependent cellular cytotoxicity (ADCC) to SARS-CoV-2 spike protein expressed on cells *in vitro*, while completely lacks virus-neutralization activity. In animal models, CV804 suppressed disease progression caused by SARS-CoV-2 infection. Structural studies using HDX-MS combined with reactivity analysis with point mutants of recombinant spike proteins revealed that CV804 binds to a unique conformational epitope within the S2 domain of the spike proteins that is highly conserved among various coronaviruses. Overall, obtained data suggest that the non-neutralizing CV804 antibody recognizes the conformational structure of the spike protein displayed on the surface of infected cells and weakens the viral virulence by supporting the host immune cells’ attack through ADCC activity *in vivo*. The CV804 epitope information revealed in this study is useful for designing pan-corona antibody therapeutics and universal coronavirus vaccines for preparing potential future pandemics.

## Introduction

The COVID-19 pandemic, caused by the SARS-CoV-2 beta coronavirus discovered in 2019, has had a severe impact on the global health and economy [1]. Besides COVID-19, various coronaviruses have caused a range of infectious diseases likely through animal-to-human spillover, posing a significant threat to public health. Coronaviridae contains four genera based on antigenicity and genetic criteria: alpha, beta, gamma, and delta [2]. Common human coronaviruses, such as HCoV-OC43, HCoV-HKU1 (beta coronaviruses), and HCoV-229E and HCoV-NL63 (alpha coronaviruses), are known to circulate annually and cause mild to moderate upper respiratory tract diseases [3, 4]. In addition, the severe acute respiratory syndrome coronavirus (SARS-CoV) that emerged in 2002 to 2003 and the Middle East respiratory syndrome coronavirus (MERS-CoV) that emerged in 2012 are representative examples of highly pathogenic coronaviruses that caused pandemics with high mortality rates [5]. These historical data strongly suggest that zoonotic spillover of coronaviruses will likely occur in the future [6]. Therefore, preparing for possible next pandemic by developing broad-spectrum antiviral drugs and vaccines is a pressing need and proactive measure against coronavirus outbreaks.

Anti-virus spike antibodies show promise as effective drugs capable of promptly addressing new coronavirus-derived infections in case of future pandemics; virus neutralization, preventing virus entry into the host cells, is the most widely pursued mechanism for antibody drugs. However, the high specificities of antibody therapeutics prevent their common use to treat different coronaviruses and easily allow virus-mutation to escape from the antibodies [7]. Most of the neutralizing antibodies (nAbs) reported against SARS-CoV-2, SARS-CoV-1, and MERS-CoV target the receptor-binding domain (RBD) in the S1 domain, inhibiting the virus from binding to target cells. However, obtaining anti-RBD antibodies with broad reactivity against diverse viruses and overcoming the escape mutations pose difficulties [8–12]. On the other hand, the S2 domain shows a higher degree of conservation across various coronaviruses than the S1 domain. Although the expected broad cross-reactivity makes the S2 domain attractive target for developing antibody drugs against multiple coronaviruses [13], neutralization limits the targetable antibody epitope regions to a few regions within the S2 domain. Indeed, existing neutralizing antibodies targeting S2, such as B6 and S2P6, bind to the restricted membrane-proximal regions and work by preventing the refolding of the S2 subunit, thereby inhibiting membrane fusion, an essential event for the virus to introduce the viral genome into the host cell cytoplasm for replication [14–18]. Antibodies to another S2 epitope such as 76E1 have also been reported to inhibit S2’ cleavage and membrane fusion, thereby demonstrating neutralizing activity [19].

Antibodies targeting a less variable region outside the RBD, specifically the S2 domain in the virus spike, have not undergone extensive validation as pharmaceuticals due to their limited *in vitro* infection-blocking activity (virus neutralization). Nonetheless, even antibodies lacking neutralizing activity that are induced in infected patients may support the host immune mechanisms [13] such as antibody-dependent cellular cytotoxicity (ADCC), contributing to favorable patient outcomes and preventing infection from related viruses [20–22].

Therefore, in this study, we expanded and explored targetable regions for therapeutic antibodies in the less mutable S2 domain. We hypothesized that the intrinsic ADCC function of an antibody is significant for the therapeutical capability even without virus-neutralization. Several recent reports support our approach [13, 22].

We successfully identified CV804, a cross-reactive antibody called against representative strains of beta coronaviruses. CV804 showed ADCC activity and inhibited the exacerbation of disease caused by viral infection despite lacking neutralizing activity. Structural and functional analysis revealed that CV804 targets a unique epitope within the highly conserved S2 domain of the spike protein. Interestingly, the accessibility of antibody to this epitope depends on the dynamic structural changes of the spike. Identifying such highly conserved epitopes holds promise for the design of broad-spectrum coronavirus vaccines and antiviral agents against current and future emerging SARS-CoV-2 mutants and other coronavirus genera.

## Materials and methods

### Plasmids

Expression plasmids for full-length spike proteins of SARS-CoV-2 or other corona-related viruses were constructed for DNA-immunization and binding assay by flow cytometry. Amino acid sequence of each spike protein was codon-optimized and its cDNA was synthesized (Azenta), and the cDNA fragment was inserted into the pcDNA 3.1 Hygro(+) vector (ThermoFisher Scientific) with fragments of IRES and cDNA of TagBFP (evrogen). GISAID IDs of SARS-CoV-2 mutant strains used were: alpha, EPI_ISL_768526; gamma, EPI_ISL_833366; delta, EPI_ISL_2158617; lambda, EPI_ISL_4204973; theta, EPI_ISL_3869208; mu, EPI_ISL_4470503; omicron BA1, EPI_ISL_6640917; BA2 EPI_ISL_9595859; BA5, EPI_ISL_13241867; BQ1.1, EPI_ISL_15579783; XBB, EPI_ISL_15669344; XE, EPI_ISL_12703378, respectively. The GenBank accession numbers of spike proteins of various corona viruses are (simple names that shown in the Figures in parenthesis): SARS-CoV-2, QHD43416.1; SARS-CoV, P59594; MERS-CoV, YP_009047204.1; HCoV-HKU1, YP_173238.1; HCoV-NL63, YP_003767.1; HCoV-229E, NP_073551.1; Bat-coronavirus (Bat-CoV), QHR63300.1; Bat Hp-beta coronavirus/Zhejiang2013 (Bat Hp-b-CoV), NC_025217.1; Rousettus bat coronavirus HKU9 (Bat HKU9), EF065513; Tylonycteris bat coronavirus HKU4 (Bat HKU4), NC_009019.1; Bovine coronavirus strain L9 (Bovine L9), P25191.1; Murine hepatitis virus strain A59 (MHV), AY700211.1; Pangolin coronavirus_GX-P1E (PCoV_GX-P1E), MT040334.1; bat SARS coronavirus HKU3-3 (Bat HKU3-3), DQ084200.1; Bat SARS coronavirus HKU3-12 (Bat HKU3-12), GQ153547.1; BtRs-BetaCoV/GX2013 (BtRs-BetaCoV), KJ473815.1; Swine acute diarrhea syndrome coronavirus (SADS-CoV), AVM41569.1; China Rattus coronavirus HKU24 (ChRCoV HKU24), YP_009113025.1; Betacoronavirus Erinaceus/VMC/DEU/2012 (EriCoV), YP_009513010.1; Pipistrellus bat coronavirus HKU5 (Pi-BatCoV_HKU5), ABN10875.1; Rousettus bat coronavirus (Ro-BatCoV), AOG30822.1; Longquan Aa mouse coronavirus (Longquan Aa mCoV), AID16631.1; Eidolon bat coronavirus/Kenya/KY24/2006 (Eidolon BatCoV), ADX59466.1; Rodent coronavirus (Rodent CoV), YP_009755834.1; Rhinolophus bat coronavirus HKU2 (Rhinolophus bat CoV HKU2), YP_001552236.1; NL63-related bat coronavirus (NL63-related BatCoV), YP_009824967.1; Wigeon coronavirus HKU20 (Wigeon CoV HKU20), YP_005352871.1; Bulbul coronavirus HKU11-934 (Bulbul CoV HKU11), YP_002308479.1; Porcine epidemic diarrhea virus, AGO58924.1; Munia coronavirus HKU13-3514 (Munia CoV HKU13), YP_002308506.1; Beluga whale coronavirus SW1 (Beluga whale CoV SW1), YP_001876437.1; Avian coronavirus IA1162/2020 (Avian coronavirus), QOS02275.1 (Table 1). Point mutant-expression plasmids of SARS-CoV-2 spike protein were made by conventional mutagenesis using overlap extension PCR.

**Table 1:** Accession numbers of spike proteins of SARS-CoV-2 and related coronavirus.

Virus name (NCBI)	Accession#	Category of coronavirus
SARS-CoV-2	QHD43416.1	β-CoV
SARS-CoV	P59594	β -CoV
MERS-CoV	YP_009047204.1	β -CoV
HCoV-HKU1	YP_173238.1	β -CoV
HCoV-NL63	YP_003767.1	α-CoV
HCoV-229E	NP_073551.1	α-CoV
Bat-coronavirus	QHR63300.1	β-CoV
Bat Hp-beta coronavirus/Zhejiang2013	NC_025217.1	β-CoV
Rousettus bat coronavirus HKU9	EF065513	β-CoV
Tylonycteris bat coronavirus HKU4	NC_009019.1	β-CoV
Bovine coronavirus strain L9	P25191.1	β-CoV
Murine hepatitis virus strain A59	AY700211.1	β-CoV
Pangolin coronavirus_GX-P1E	MT040334.1	β-CoV
Bat SARS coronavirus HKU3-3	DQ084200.1	β-CoV
Bat SARS coronavirus HKU3-12	GQ153547.1	β-CoV
Swine acute diarrhea syndrome coronavirus	AVM41569.1	β-CoV
China Rattus coronavirus HKU24	YP_009113025.1	β-CoV
Betacoronavirus Erinaceus/VMC/DEU/2012	YP_009513010.1	β-CoV
Pipistrellus bat coronavirus HKU5	ABN10875.1	β-CoV
Rousettus bat coronavirus	AOG30822.1	β-CoV
Longquan Aa mouse coronavirus	AID16631.1	β-CoV
Eidolon bat coronavirus/Kenya/KY24/2006	ADX59466.1	β-CoV
Rodent coronavirus	YP_009755834.1	β-CoV
BtVs-BetaCoV/SC2013	AHY61337.1	β-CoV
Rhinolophus bat coronavirus HKU2	YP_001552236.1	α-CoV
NL63-related bat coronavirus	YP_009824967.1	α-CoV
Wigeon coronavirus HKU20	YP_005352871.1	δ-CoV
Bulbul coronavirus HKU11-934	YP_002308479.1	δ-CoV
Porcine epidemic diarrhea virus	AGO58924.1	δ-CoV
Munia coronavirus HKU13-3514	YP_002308506.1	δ-CoV
Beluga whale coronavirus SW1	YP_001876437.1	γ-CoV
Avian coronavirus IA1162/2020	QOS02275.1	γ-CoV

### Cells

HEK293T cells and mouse P3U1 myeloma cells were sourced from the American Type Culture Collection. VeroE6/TMPRSS2 cells obtained from the National Institutes of Biomedical Innovation (Tokyo, Japan) were used to evaluate the antiviral activity against SARS-CoV-2. HEK293T and VeroE6/TMPRSS2 cells were maintained in Dulbecco’s modified Eagle’s medium (Thermo Fisher Scientific) supplemented with 10% heat-inactivated fetal bovine serum (FBS) at 37°C with 5% CO2. P3U1 cells cultured in Iscove’s modified DMEM (IMDM) (Thermo Fisher Scientific) supplemented with 10% FBS. ExpiCHO cells and Expi293F cells were purchased (Thermo Fisher Scientific) and maintained in ExpiCHO expression medium (Thermo Fisher Scientific) or in Expi293 expression medium (Thermo Fisher Scientific) at 37°C under 8% CO2, respectively.

### SARS-CoV-2 clinical isolates

SARS-CoV-2 clinical isolates were obtained from the National Institute of Infectious Diseases (NIID; Tokyo, Japan): hCoV-19/Japan/TY/WK-521/2020 (Pango Lineage: A), hCoV-19/Japan/QK002/2020 (B.1.1.7), hCoV-19/Japan/QHN001/2020 (B.1.1.7), hCoV-19/Japan/QHN002/2020 (B.1.1.7), hCoV-19/Japan/TY7-501/2021 (P.1), hCoV-19/Japan/TY8-612/2021 (B.1.351), hCoV-19/Japan/TY11-927/2021 (AY.122), hCoV-19/Japan/TY33-456/2021 (C.37), hCoV-19/ Japan/TY28-444/2021 (P.3), hCoV-19/ Japan/TY38-873/2021 (BA.1.18), hCoV-19/ Japan/TY38-871/2021 (BA.1.1), hCoV-19/ Japan/TY40-385/2022 (BA.2), hCoV-19/ Japan/TY41-721/2022 (BA.2.12.1), hCoV-19/ Japan/TY41-716/2022 (BA.2.75), hCoV-19/ Japan/TY41-703/2022 (BA.4.1), hCoV-19/ Japan/TY41-763/2022 (BA.4.6), hCoV-19/Japan/TY41-702/2022 (BE.1), hCoV-19/ Japan/TY41-704/2022 (BA.5.2.1), hCoV-19/ Japan/TY41-796/2022 (BQ.1.1), hCoV-19/ Japan/TY41-795/2022 (XBB.1), hCoV-19/ Japan/TY41-686/2022 (XE), hCoV-19/ Japan/TY41-820/2022 (BF.7), hCoV-19/Japan/TY41-828/2022 (BF.7.4.1), hCoV-19/ Japan/23-018/2022 (XBB.1.5), and hCoV-19/Japan/TY7-503/2011 (P.1). SARS-CoV-2 strains were propagated in VeroE6/TMPRSS2 cells, and infectious titers were determined using the standard tissue culture infectious dose (TCID)50 method in VeroE6/TMPRSS2 cells.

### Production of mAbs

Anti-S2 mouse monoclonal antibodies were produced by a conventional hybridoma method [23]. In brief, A/J mice were immunized 5–6 times with 20 μg of SARS-CoV-2 full-length spike protein expression plasmid DNA as described previously [18]. Recombinant S2 protein or 293T cells transfected with the spike protein expression plasmid were i.d. injected for the final boost immunization. Three days after the final boost, the spleen cells were fused with P3U1 myeloma cells as described previously [18]. After hypoxanthine-aminopterin-thymidine (HAT) selection, the hybridomas were screened for secretion of specific mAbs with flow cytometry using 293T cells transiently transfected with the expression plasmid encoding SARS-CoV-2 full-length spike protein. After multiple rounds of cell cloning by limiting dilution, the established hybridomas were grown to harvest the mAbs from cell culture supernatants. The isotype of the mAbs was determined using a mouse immunoglobulin isotyping Cytometric Bead array kit (BD Pharmingen).

### Antibody preparation

Fv sequences of CV804 were determined by RACE PCR using Smarter RACE 5’/3’ kit (Clontech) using mRNA isolated from the hybridoma. Variable heavy and light genes were inserted into custom plasmids that encode the constant region for the human IgG1 heavy chain and respective lambda and kappa light chains (pcDNA3.4 TOPO vector, Thermo Fisher Scientific) (Table 2). The antibodies were expressed in Expi293F cells by co-transfecting with the two plasmids encoding corresponding heavy chain or light chain using Expifectamine transfection reagent (Thermo Fisher Scientific) according to the manufacturer’s instructions. The recombinant antibodies expressed in the culture supernatants were purified with Protein A agarose. The purified antibodies were analyzed by SDS-PAGE.

**Table 2:** Antibody sequence list.

Antibody	Heavy chain	Light chain
REGN10987	QVQLVESGGGVVQPGRSLRLSCAASGFTFSNYAMYWVRQAPGKGLEWVAVISYDGSNKYYADSVKGRFTISRDNSKNTLYLQMNSLRTEDTAVYYCASGSDYGDYLLVYWGQGTLVTVSS	QSALTQPASVSGSPGQSITISCTGTSSDVGGYNYVSWYQQHPGKAPKLMIYDVSKRPSGVSNRFSGSKSGNTASLTISGLQSEDEADYYCNSLTSISTWVFGGGTKLTVLGQPKAA
LY-CoV 1404	QITLKESGPTLVKPTQTLTLTCTFSGFSLSISGVGVGWLRQPPGKALEWLALIYWDDDKRYSPSLKSRLTISKDTSKNQVVLKMTNIDPVDTATYYCAHHSISTIFDHWGQGTLVTVSS	QSALTQPASVSGSPGQSITISCTATSSDVGDYNYVSWYQQHPGKAPKLMIFEVSDRPSGISNRFSGSKSGNTASLTISGLQAEDEADYYCSSYTTSSAVFGGGTKLTVLGQPKAA

### ELISA

The mouse antibodies produced in this study were assessed with the reactivity in an ELISA with SARS-CoV-2 trimeric spike proteins (ACROBio SPN-C52H9), monomeric S2 protein (ACROBio S2N-C52H5), and trimeric S2 protein (home-made using S2 region corresponding to 686–1208 amino acids of SARS-CoV-2 spike protein). In a separate experiment, antibody cross-reactivity to HCoV-OC43 was assessed in a similar ELISA using S2 domain of the HCoV-OC43 (Sino Bio 40607-V08B1). All the virus proteins are His-tagged. In the ELISA, 100 ng/50 μL/well of the tested mouse antibodies were indirectly coated to the plates that had been pre-coated with anti-mouse IgG antibodies (Jackson Immunoresearch 115-005-071). After three washes, dilutions of the spike proteins were added and incubated at room temperature for 1 hour. The His-tagged spike proteins captured by the test antibodies were detected with an anti-histidine tag human antibody (home-made) followed by alkaline phosphatase-labeled anti-human IgG Fcγ antibody (Jackson Immunoresearch 109-055-098) and P-nitrophenyl phosphate substrate. EC_50_ values were determined by plotting the compound concentration versus inhibition and fitting the data using a four-parameter logistical fit (Model 205, XLfit, IDBS).

### In-cell SARS-CoV-2 ELISA

To detect SARS-CoV-2 infection, cells were distributed at 3x10^4^ cells/well in a 96-well plate and were exposed to a virus solution at 100 μL/well. After incubation at 37°C for 1 hour, the medium was replaced with a 2xMEM/8% FBS and 2% MC mixture in a 1:1 ratio, and later with 1xMEM/4% FBS/1% MC at 200 μL/well. The cells were incubated at 37°C for 16–17 hours, the medium was removed, leaving 30 μL in each well, then washed with PBS. A 4% paraformaldehyde solution was added at 300 μL/well and left at room temperature for 30 minutes. Ethanol wiped the plate, retaining about 30 μL, before 4% paraformaldehyde removal. The cells were twice washed with PBS and then with wash buffer, leaving behind around 50 μL. The cells were then treated with a 2x permeability buffer (0.45 g Glycine, 145.5 ml PBS, 0.75 ml Triton X-100) for 10 minutes at room temperature and washed thrice with wash buffer, retaining 50 μL after each wash. A 2% BSA solution, prepared with PBS, was added and left to incubate at room temperature for 30 minutes. The primary antibody (hCV804-40, LY-CoV 1404, REGN10987, Human IgG1 (BE0297, Bio X Cell), anti-SARS-CoV-2 Nucleocapsid antibody (Clone 1035111)) was added and left for 2 hours at room temperature before four wash buffer rinses. The secondary antibody diluted in 0.1% BSA solution was added, followed by an hour of incubation and four washes. TrueBlue reagent, which is a peroxidase substrate, was added for staining visualization, and infected foci were enumerated using Immunospot (Cellular Technology Limited).

### Flow cytometry and data analysis

293T cells were transfected with the various spike protein expression plasmids. Two days after transfection, the cells were harvested, stained with anti-SARS-CoV-2 mAbs, followed by PE-labeled anti-mouse IgG (Jackson ImmunoResearch, 115-116-146) or PE-labeled anti-human IgGs (Jackson ImmunoResearch, 109-116-170). The stained cells were analyzed with a LSRFortessa X20 flow cytometer (BD Biosciences). Data analysis was performed using FlowJo software. Because the bicistronic plasmids used in this study contain internal ribosomal entry site (IRES) and subsequent TagBFP cDNA in addition to the different spike proteins, TagBFP was expressed with the same mRNA encoding the spike proteins. Therefore, in the flow cytometry data TagBFP-expressing cell population was gated as the antigen-expressing cell population. The logarithm of the median fluorescent intensity (logMFI) of the PE signal in the gated population was taken, subtracted by the arbitrary of value 1.5 roughly representing cell autofluorescence level, and shown in heatmap figures. EC_50_ values were determined by plotting the compound concentration versus inhibition and fitting data with a four-parameter logistical fit (Model 205, XLfit).

### Neutralizing antiviral activity

Antiviral activities against SARS-CoV-2 were evaluated using VeroE6/TMPRSS2 cells. The viral strains hCoV-19/Japan/TY/WK-521/2020 and SARS-CoV-2/Japan/WK-521/2020 were employed, utilizing VeroE6/TMPRSS2 cells as the infected cells. The culture medium consisted of MEM supplemented with 2% FBS. For antibody dilution, CV804, REGN10987, LY-CoV1404 and isotype control antibody were adjusted to a maximum concentration of 3.3 μM, each antibody with a 3-fold serial dilution of 10 points. The virus was adjusted to a concentration of 3000 TCID_50_/well. The adjusted antibody dilutions and virus solution were mixed in equal proportions and allowed to react at room temperature for 1 hour. The virus-antibody mixture was then added to VeroE6/TMPRSS2 cells at a density of 15,000 cells/well and cultured at 37°C, under 5% CO2. After 72 hours, CellTiter-Glo 2.0 (Promega) was added to measure the fluorescence intensity. Antiviral activity against SARS-CoV-2 was assessed by monitoring the cell viability. EC_50_ values were determined by plotting the compound concentration versus inhibition and fitting the data using a four-parameter logistical fit (Model 205, XLfit).

### Measurement of ADCC activity

As target cells used in ADCC assay, we established a cell line that expressed recombinant CoV-2 spike protein and the expression was inducible by adding tetracycline in the cell culture. In brief, a DNA fragment encoding SARS-CoV-2 spike full length, IRES and cDNA of TagBFP was inserted into pcDNA5/FRT/TO vector (Invitrogen). Flp-In T-REx-293 cells (Invitrogen) were co-transfected with the constructed plasmid and pOG44 plasmid for recombinase-expression (Invitrogen), cultured in presence of hygromycin for two weeks and cloned by limiting dilution method to establish a cell line, SJ147. The tetracycline-inducible expression of SARS-CoV-2 S was confirmed by flow cytometry.

ADCC activity was measured by mouse FcγRIV ADCC Reporter Bioassay kit (Promega) according to manufacturer’s instruction. SJ147 cells were seeded in 96-well white culture plates with cell culture medium containing 1 ug/ml tetracycline and incubated in 5% CO_2_ incubator overnight. Mouse antibodies (CV801, CV804, CV820, CV925, CV1117) and effector cells (Promega) expressing mouse FcγRIV and NFAT luciferase reporter were added to the cells, and the reaction was conducted at 37°C in a CO2 incubator for 6 hours. Next, BioGlo reagent (Promega) was added and allowed to react for 5 minutes. Luminescence was measured by Enspire plate reader (Perkin Elmer).

### Mouse experiments and approvals

Mouse study was conducted under applicable laws and guidelines. The protocols received approval from the Shionogi Pharmaceutical Research Centre Institute Director (Shionogi & Co., Ltd., Toyonaka, Japan) based on the report of the Institutional Animal Care and Use Committee (Assurance number: S21043D). Mice were handled by experienced personnel to alleviate stress and suffering to the animals. All mice were kept under specific pathogen-free conditions, with regulated temperature and a 12-hour light and dark cycle. They had access to water and standard laboratory chow ad libitum, and the humidity was maintained at 30–70%. For each treatment group, 5 mice were housed per cage, with chip paper bedding and a wooden bite stick for environmental enrichment. Virus inoculations were performed under anesthesia by intramuscular administration of an anesthetic solution containing medetomidine hydrochloride, midazolam, and butorphanol tartrate, with measures taken to minimize animal suffering. The *in vivo* studies were not conducted in a blinded manner, and animals were randomly assigned to infection groups. Sample size calculations were not performed for each study; instead, sample sizes were determined based on previous *in vivo* virus challenge experiments. Female BALB/c mice 35 to 45 weeks old (n = 5) were inoculated via the intranasal route with 1.0×10^5^ TCID_50_/mouse (in 50 μL) of SARS-CoV-2 hCoV-19/Japan/TY7-501/2021 (gamma strain). Immediately after infection, the mice were intravenously administered 40 mg/kg of CV804, CV804 (LALA), REGN10987 or isotype control mAb (C1.18.4). As a reference, PBS-inoculated uninfected control mice were also prepared. Body weight and survival were monitored once daily until day 6 post-virus infection. At 6 days post-infection, the mice were euthanized via cervical dislocation under isoflurane anesthesia. If the mice had lost more than 20% of their body weight compared to their initial body weight according to humane endpoints, they were immediately euthanized and regarded to be dead in the analysis for survival time. The numbers of mice that survived, were euthanized according to humane endpoints, or died before reaching the humane endpoints are summarized in S1 Table.

### HDX-MS analysis

The hydrogen/deuterium exchange method has been previously described [24, 25]. The system for HDX-MS experiments used the HDX-PAL system for deuterium labeling (Leap Technologies Inc.), an enzyme pepsin column for sample digestion (Waters), a HyperSil Gold column as a trap column (Thermo Fisher Scientific), an Acclaim PepMap 300 C18 column as an analytical column (Thermo Fisher Scientific), and Orbitrap Eclipse for mass measurement of digested peptides (Thermo Fisher Scientific). The target protein for hydrogen/deuterium exchange epitope mapping was a chimeric protein of the spike protein of SARS-CoV-2 expressed in HEK293 cells. The chimeric protein used included the ectodomain of the spike protein of SARS-CoV-2 (amino acids 1–1213 of the S protein described in Genbank ACC No. QHD43416.1), with amino acids 986 (K) and 987 (V) replaced with proline (P). The deuterated buffer was prepared at pH 7.4 using 10 mM PBS buffer adjusted with D2O. The above target protein was allowed to react at 37°C for 30 minutes in the presence or absence antibody. The initiation of deuterium labeling, control of reaction time, quenching reaction, injection into the UPLC system, and control of digestion time were all carried out automatically by the HDX-PAL system. First, the target protein/antibody complex or target protein was diluted 10-fold with deuterated buffer to initiate deuterium labeling. This was carried out under conditions of 10°C for 60, 120, and 240 seconds of labeling time. The deuterium-labeled sample (27 μL) was mixed with an equal volume of quenching buffer (4 mol/L guanidine hydrochloride, 0.2 mol/L glycine hydrochloride, 0.5 mol/L TCEP) and quenched at 0°C for 3 minutes. Subsequently, the quenched sample (50 μL) was injected into an Enzyme Pepsin Column for online pepsin digestion. The digested peptides were trapped on a trap column (Hypersil Gold column) at 1°C and eluted onto an analytical column (Acclaim PepMap300 C18) using a 7-minute gradient separation of 10%-35% B (mobile phase A: 0.1% formic acid in water, mobile phase B: 0.1% formic acid/acetonitrile). Mass spectrometry (Orbitrap Eclipse) was set with an electrospray voltage of 4000 V, a cycle time of 1 second, and a mass/charge range of 260–2000 in positive ion mode to acquire both full-scan and MS/MS spectra. To identify the peptides from the acquired mass spectra, a search was conducted against a database containing amino acid sequences of the spike protein of SARS-CoV-2 using Byos software (Protein Metrics). The retention time and mass information of the identified peptides were then imported into HDExaminer software (Sierra Analytics) to automatically calculate the deuterium exchange rates of each peptide in the hydrogen/deuterium exchange experiment. Peptides with calculated deuterium exchange rates were filtered according to the criterion that the difference in deuterium exchange rates (ΔD%) between the antibody-present and -absent samples of two or more adjacent peptides was 5% or more. The amino acid residues of the spike protein of SARS-CoV-2 corresponding to the region containing the peptides meeting the criterion were identified as the epitope of the antibody.

## Results

### Antibody isolation and binding activity to S protein

**Fig 1:**
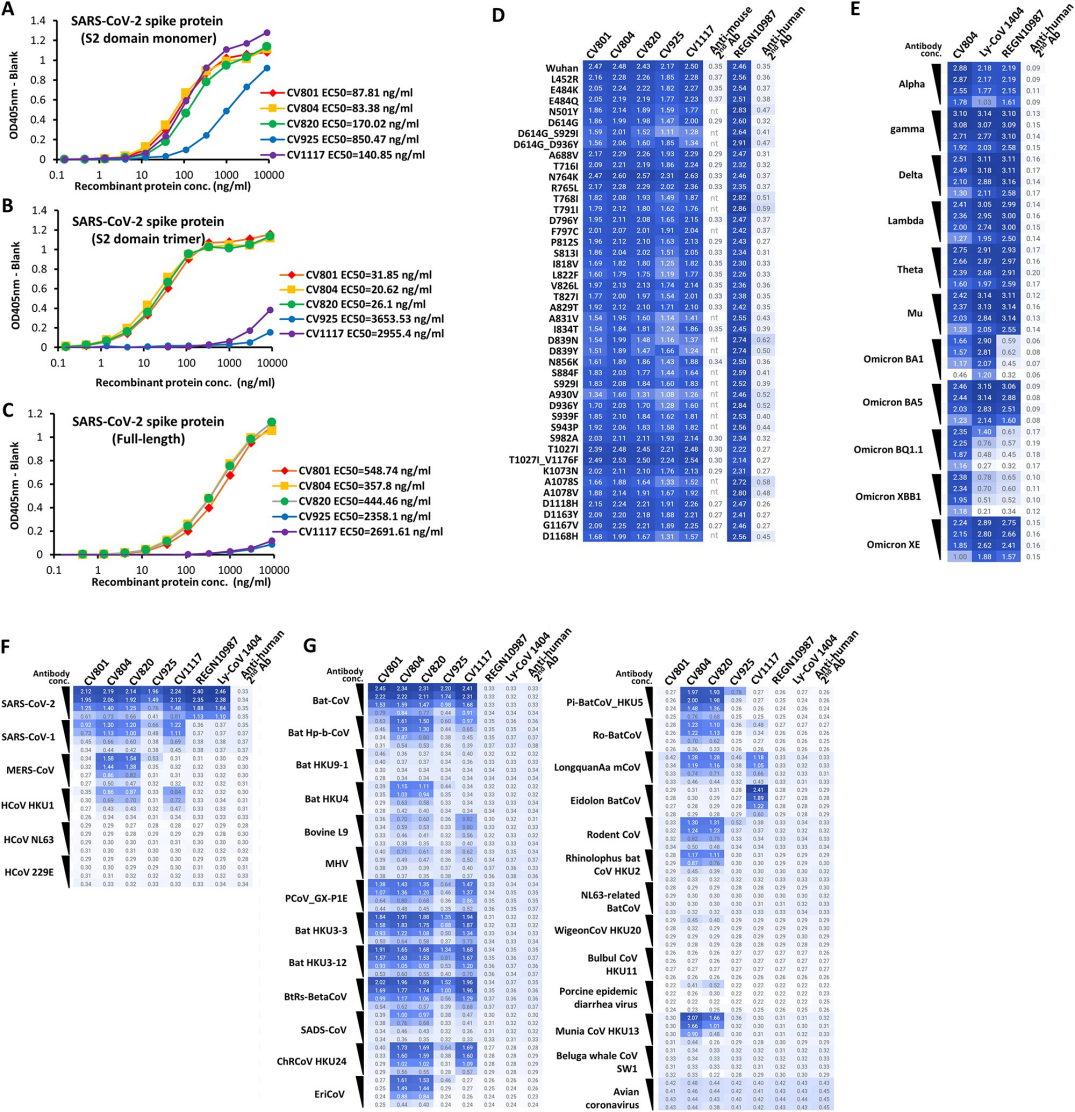
Binding activity of anti-SARS-CoV-2 S protein mAbs. A-C show binding of five mAbs in ELISA to three purified S proteins; the soluble S2 domain monomer (A), trimer (B), and full-length (C). Representative data of two independent experiments are shown. D-F show reactivity of the mAbs in flow cytometry using 293T cells transfected with full-length S protein expression plasmids. The cells were stained by 1 μg/ml (D) or ten-fold serial dilution from 0.01–10 nM of each mAb (E-G), followed by PE-labeled secondary antibodies. Binding to SARS-CoV-2 S proteins with a point mutant (D), S proteins of SARS-CoV-2 mutant strains (E), S proteins of human corona viruses (F), or S proteins of other corona-related viruses (G) is depicted in heatmaps of logMFI of PE signals.

To obtain antibodies that effectively bind to a wide range of coronaviruses, we targeted S2 subunit of the spike protein of SARS-CoV-2. For immunization, plasmid DNAs encoding full-length SARS-CoV-2 S protein or S2 domain, full-length SARS-CoV-2 S protein or S2 domain-expressing cells or purified full-length SARS-CoV-2 S protein or S2 domain were used. Using the antiserum, we confirmed their binding to the S2 region and various coronaviruses, including MERS-CoV and SARS-CoV. To obtain mAbs which react with the native form of SARS-CoV-2 S protein, hybridomas were screened by binding to full-length spike protein and S2 domain of SARS-CoV-2 expressed on 293T cells by flow cytometry. We eventually obtained five antibodies, CV801, CV804, CV820, CV925 and CV1117. Their immunoglobulin subclass was all mouse IgG2a kappa. Their reactivity with S2 protein monomers, trimers, and full-length S protein trimers in ELISA is shown in Fig 1.

Remarkably, all the five mAbs significantly reacted with S2 monomer (Fig 1A). Interestingly, three antibodies (CV801, CV804, and CV820) displayed stronger affinity for the trimer compared to the monomer and CV925 and CV1117 showed very low affinity with both S2 trimer and full-length proteins (Fig 1A–1C), suggesting that the structures of these purified proteins are different from those expressing on the cells. For this reason, we tested mAbs for the reactivity mainly by cell ELISA or flow cytometry using antigen-expressing cells. Next, we examined their reactivity with single mutations of the SARS-CoV-2 S protein, which have been identified globally. 293T cells were transiently transfected with the plasmids expressing CoV-2 S protein with various single mutants, and subjected to flow cytometry analysis. All the five mAbs reacted with all the cells with single mutation tested (Fig 1D). To assess the cross-reactivity of these anti-S2 mAbs with S proteins of 11 SARS-CoV-2 mutant strains, 7 human coronaviruses and 26 other corona-related viruses. Flow cytometry was performed using 293T cells transfected with expression plasmids for full-length S proteins from SARS-CoV-2 mutant strains (Fig 1E) or different types of coronaviruses (Fig 1F and 1G). CV804 recognized all eleven SARS-CoV-2 mutant strains tested while anti-S1 mAbs did not bind to some of Omicron strains (Fig 1E). Five mAbs exhibited broad cross-reactivity to these S proteins whereas anti-S1 antibodies, REGN10987 and LY-CoV 1404 did not react with any other S proteins than SARS-CoV-2 at all. Especially, CV804 and CV820 reacted with S proteins of 3 other human coronaviruses than SARS-CoV-2 (Fig 1F), and with 18 corona-related viruses (Fig 1G), As for another human coronavirus HCoV-OC43, we performed ELISA using recombinant protein (S1 Fig). Binding of CV804 to HCoV-OC43 was observed in a dose-dependent manner. Thus, mAbs, which binds to the S2 domain of the viral spike protein, exhibits resistance to mutations, allowing it to effectively react with various mutant strains, including the Omicron strains, as well as numerous related coronaviruses [26]. Based on the broad cross-reactivity and highest affinity of CV804 among five mAbs, we selected CV804 for further characterization.

### CV804 binds to SARS-CoV-2 and its variants but does not neutralize them

**Fig 2:**
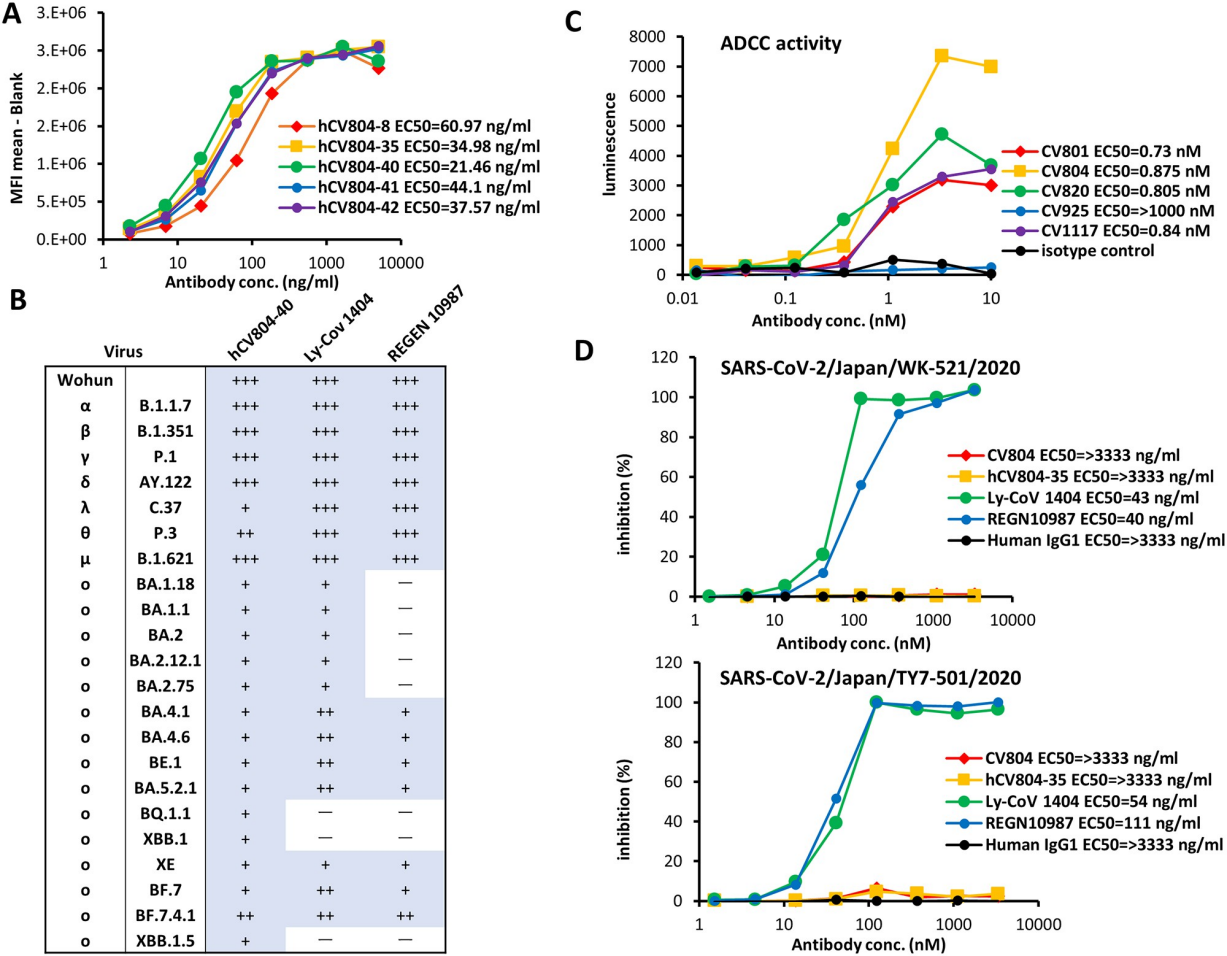
CV804 binds to SARS-CoV-2 and its variants, but does not neutralize them. (A) Five humanized CV804 antibodies were analyzed by flow cytometric assay of titrated candidates against SARS-CoV-2 spike protein expressing cells. Four parameter logistic curve fitted with EC_50_ in parentheses. Normalized by secondary alone and saturation signal. Representative data of two independent experiments are shown. (B) Evaluation of hCV804-40 antibody, REGN10987, and LY-CoV1404 antibody binding to cells infected with SARS-CoV-2 variants. "+++", "++", "+" indicate positive binding; "-" indicates negative binding. (C) Evaluation of antibody-dependent cell-mediated cytotoxicity ADCC activity against spike-expressing cells for mouse derived antibodies. (D) *In vitro* cell infection inhibition experiments were conducted. The viral strains used were hCoV-19/Japan/TY/WK-521/2020 (top) and SARS-CoV-2/Japan/TY-501/2020 (bottom), with VeroE6/TMPRSS2 cells being used as the target cells. The hCV804-35 antibody, REGN10987, and LY-CoV 1404 were measured in this experiment. The antibody concentration that inhibited cell death by 50% was defined as the IC_50_.

For therapeutic purposes in humans, Fv sequences of the mouse antibody CV804 (IgG-κ) was determined and humanized using our proprietary algorithm (Shionogi Co. Ltd., Osaka, Japan). The variable regions were humanized based on the Kabat antibody numbering scheme, with substitutions of amino acids guided by physical property predictions from the closest germline and known human antibody sequences and structures. Consequently, the humanized antibody named hCV804 was generated. This antibody was produced and assessed for binding to the S protein. Remarkably, the humanized antibody maintained binding activity to the S protein, with a similar Kd value to that of the mouse-derived CV804 (Fig 2A).

To gain deeper insight into the characteristics of CV804, we evaluated different variants of SARS-CoV-2 virus-infected cells, particularly the Omicron variant, using in-cell SARS-CoV-2 ELISA or flow cytometry to assess their binding efficacy to spike protein-expressing cells. Unlike S1 antibodies such as REGN10987 (imdevimab) [27] and LY-CoV 1404 (bebtelovimab) [28], CV804 demonstrated binding activity against multiple strains, including B.1.1.7 (alpha), B.1.351 (beta), P.1 (gamma), B.1.617.1 (kappa), B.1.617.2 (delta), and B.1.1.529 (omicron, BA.1), as well as the Omicron subvariants (BA.2.75, BA.4.1, BA.4.6, BE.1, BA.5.2.1, BQ.1.1, XBB.1, XE, BF.7, BF.7.4.1) (Fig 2B). It displayed remarkable resilience against viral mutations. We also evaluated the ADCC activity of CV804 using a reporter system and found that it had robust activity (Fig 2C). However, CV804 did not exhibit inhibitory activity against SARS-CoV-2 in a virus neutralization test using VeroE6/TMPRSS2 cells, while both REGN10987 and LY-CoV1404 exhibited strong *in vitro* antiviral activity (Fig 2D).

### Antiviral activity of non-neutralizing CV804 against SARS-CoV-2

**Fig 3:**
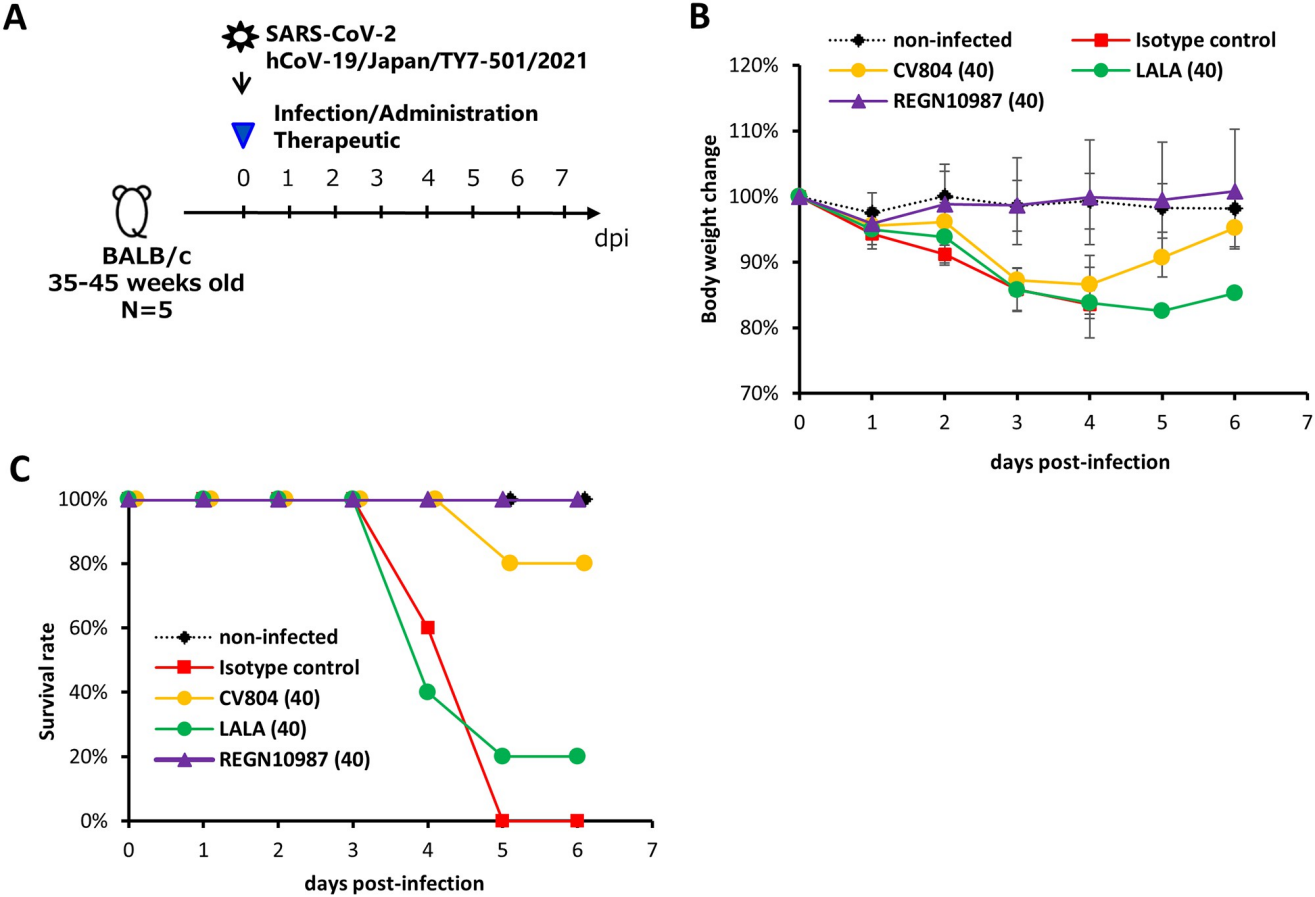
Antiviral activity of non-neutralizing CV804 against SARS-CoV-2. The therapeutic effects in aged mice were evaluated. (A) Outline for treatment protocol. Mice were intranasally inoculated with 50 μL of hCoV-19/Japan/TY7-501/2021 (1.00x10^5^ TCID50) under anesthesia. Non-infected control mice were intranasally inoculated with 50 μL of vehicle under anesthesia. Starting from immediately after virus infection, mice (n = 5/group) were intravenously administered a single dose of 40 mg/kg of CV804, CV804-LALA, and REGN10987, or a single dose of 40 mg/kg of C1.18.4 (isotype control). The body weight (B) and survival rate (C) of the mice were assessed once daily until day 6 post-virus infection.

To evaluate the therapeutic effects of CV804 *in vivo*, 35- to 45-week-old female BALB/c mice were intranasally infected with 1.0×10^5^ TCID_50_/mouse of SARS-CoV-2 hCoV-19/Japan/TY7-501/2021 (gamma strain). Immediately after infection, the mice were intravenously administered 40 mg/kg of CV804 or isotype control. All mice were observed daily for survival and body weight changes (Fig 3B and 3C). In the group treated with 40 mg/kg of CV804, the body weight of the mice decreased by approximately 15% until day 4 and recovered to a level comparable to uninfected mice by day 6 (Fig 3B). In the isotype-treated group, all mice reached the humane endpoint or died by day 5, whereas 80% of the mice in the CV804-treated group survived, indicating an improvement in the survival rate due to CV804 administration (Fig 3C). This effect was diminished on using the LALA mutant, which eliminates the Fc effector function of IgG, suggesting the possible contribution of ADCC activity to the therapeutic efficacy. However, the improvement in disease pathology observed with CV804 administration in this mouse model was limited. Although some degree of improvement was observed, the therapeutic effect was inferior to that of the neutralizing antibody (REGN10987) used as a control drug.

### The epitope of CV804 is buried within the prefusion spike trimer

**Fig 4:**
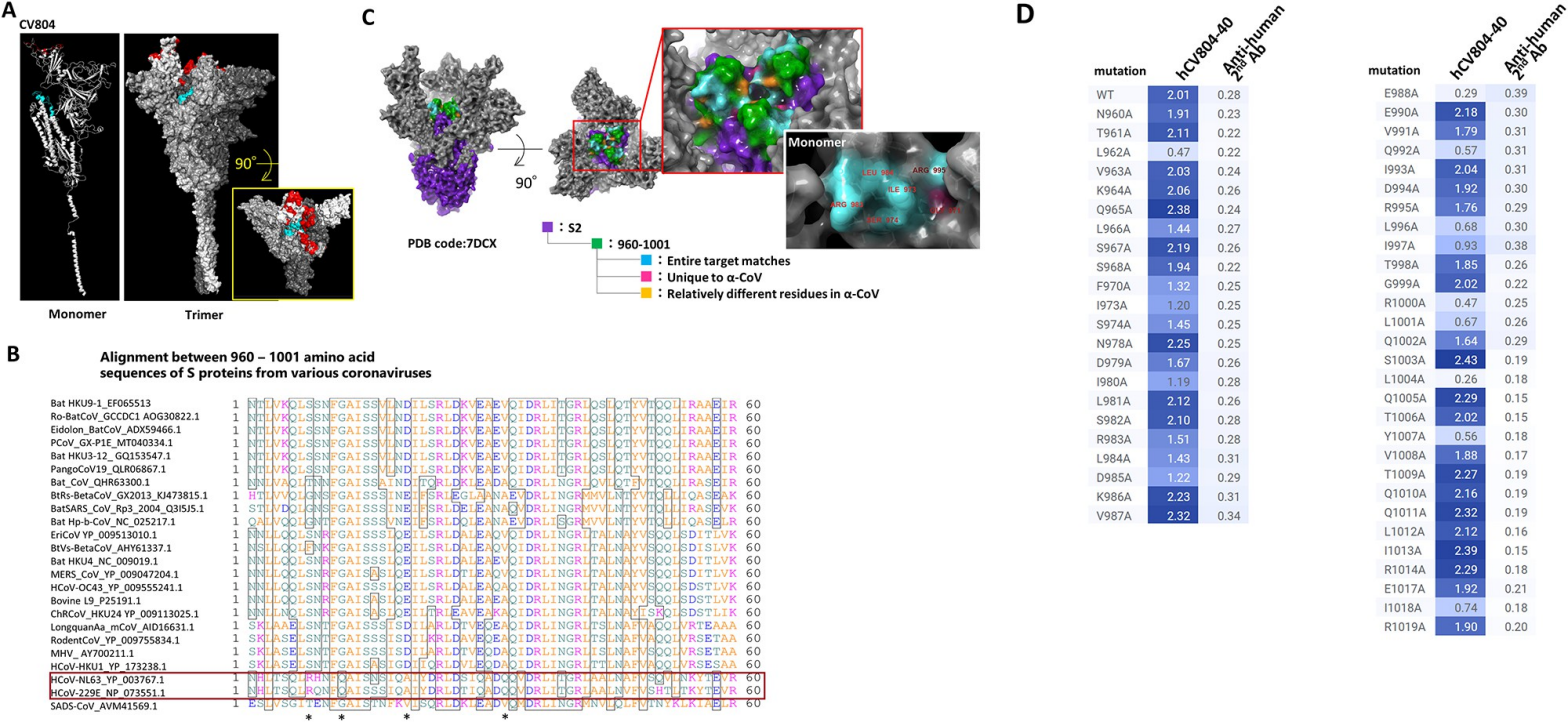
The epitope of CV804 is buried within the prefusion spike trimer. We performed epitope mapping of the CV804 antibody binding to the spike protein of SARS-CoV-2 using HDX-MS. (A) The regions detected by HDX-MS were plotted onto the three-dimensional structure (PDB ID: 6vsb_1_1_1) shown in red for the ACE2 binding recognition domain and in cyan for the regions detected by HDX-MS. The figures from left to right depict the monomer and trimer, with the figure on the right showing a top view of the spike trimer. (B) Amino acid sequence alignment of the spike protein epitope candidate region (960–1001) was performed. The red box represents alpha coronaviruses, and the asterisks indicate amino acids that are relatively different in alpha coronaviruses. (C) Highly conserved regions in alpha and beta coronaviruses were extracted and mapped onto the 2-up structure of the Wuhan strain crystal structure (PDB code: 7DCX). Purple represents the spike 2 protein, green represents the detected epitopes, cyan represents the regions that are conserved among all target coronaviruses within the detected epitopes, pink represents residues that are unique to alpha coronaviruses, and yellow represents amino acid residues that are relatively different in alpha coronaviruses. The red box enlarges the central part of the trimer. The white box illustrates the monomeric state of the region indicated by the red box, with the amino acid residue numbers indicating the universally conserved regions among coronaviruses and exposed residues on the surface of the S2 protein. (D) Reactivity loss of CV804 with SARS-CoV-2 S protein in flow cytometry by substituting alanine for an amino acid in the S protein. 293T cells were transfected with full-length CoV-2 S protein expression plasmids with a point mutation designated, stained by humanized CV804-40, followed by PE-labeled secondary antibody. Binding activity is depicted in a heatmap of logMFI of PE signal.

To further validate the conservation of the epitope recognized by CV804 and investigate the molecular basis of its broad specificity, antigen binding, and epitope interaction, we generated a spike protein ectodomain trimer and performed HDX-MS analysis to identify the CV804 epitope. We detected a peptide region within the CV804 binding trimer that showed more than 5% decrease in deuterium exchange efficiency compared to the isolated trimer, indicating the core of the epitope (Table 3). We confirmed the binding of CV804 and humanized hCV804-40 to the spike protein at positions 960–1001 (SARS-CoV-2 numbering) (Fig 4A). The data obtained, as described above, revealed the epitope region of CV804 and the specificity of the cross-reactivity of the antibody. Based on the reported higher order structure of the spike protein, we considered the existence of a rational relationship between the epitope and cross-reactivity.

**Table 3:** Epitope mapping of CV804 antibody binding to the SARS-CoV-2 spike protein using the HDX method.

Start	End	Sequence	Deuteration Time (sec)	Avg(ΔD%)	Avg(D%_#1)	Avg(D%_#2)	Avg(#D_#1)	Confidence Interval (#D_#1)	Avg(#D_#2)	Confidence Interval (#D_#2)
957	961	QALNT	60	3.30	23.81	20.52	0.71	0.17	0.62	0.16
			120	5.47	32.10	26.63	0.96		0.80	
			240	6.79	38.42	31.63	1.15		0.95	
957	962	QALNTL	60	6.26	14.29	8.03	0.57	0.18	0.32	0.82
			120	7.99	22.65	14.66	0.91		0.59	
			240	6.49	26.33	19.84	1.05		0.79	
960	970	NTLVKQLSSNF	60	11.81	38.09	26.28	3.43	1.25	2.37	0.22
			120	15.09	44.19	29.10	3.98		2.62	
			240	15.76	47.33	31.57	4.26		2.84	
960	974	NTLVKQLSSNFGAIS	60	22.41	48.84	26.42	6.35	0.62	3.44	0.65
			120	23.29	51.63	28.34	6.71		3.68	
			240	25.23	52.44	27.21	6.82		3.54	
960	977	NTLVKQLSSNFGAISSVL	60	22.73	55.29	32.56	8.85	1.42	5.21	0.22
			120	24.25	56.63	32.38	9.06		5.18	
			240	24.67	57.91	33.24	9.27		5.32	
962	969	LVKQLSSN	60	11.52	38.80	27.28	2.33	0.33	1.64	0.05
			120	15.71	45.35	29.64	2.72		1.78	
			240	17.93	49.32	31.39	2.96		1.88	
962	970	LVKQLSSNF	60	12.14	43.01	30.87	3.01	0.18	2.16	0.24
			120	13.68	47.93	34.26	3.36		2.40	
			240	13.90	50.56	36.66	3.54		2.57	
962	972	LVKQLSSNFGA	60	21.17	51.71	30.54	4.65	1.99	2.75	2.01
			120	18.30	50.76	32.45	4.57		2.92	
			240	20.12	53.92	33.79	4.85		3.04	
962	974	LVKQLSSNFGAIS	60	25.21	53.30	28.09	5.86	0.10	3.09	0.49
			120	25.60	56.61	31.01	6.23		3.41	
			240	24.36	58.13	33.77	6.40		3.72	
962	977	LVKQLSSNFGAISSVL	60	26.80	58.81	32.02	8.23	0.50	4.48	0.25
			120	26.71	60.93	34.22	8.53		4.79	
			240	25.37	61.65	36.28	8.63		5.08	
962	979	LVKQLSSNFGAISSVLND	60	25.73	59.53	33.80	9.53	2.13	5.41	0.41
			120	24.05	59.55	35.50	9.53		5.68	
			240	23.13	60.84	37.71	9.74		6.03	
963	970	VKQLSSNF	60	14.90	48.53	33.64	2.91	0.29	2.02	0.22
			120	15.35	52.88	37.53	3.17		2.25	
			240	15.39	56.18	40.79	3.37		2.45	
963	974	VKQLSSNFGAIS	60	27.66	58.04	30.38	5.80	0.93	3.04	0.63
			120	26.36	57.84	31.49	5.78		3.15	
			240	28.26	60.53	32.28	6.05		3.23	
963	977	VKQLSSNFGAISSVL	60	27.16	58.34	31.18	7.58	0.29	4.05	0.54
			120	25.27	58.82	33.55	7.65		4.36	
			240	24.27	59.66	35.39	7.76		4.60	
970	977	FGAISSVL	60	41.57	67.67	26.11	4.06	0.43	1.57	0.11
			120	38.24	65.88	27.63	3.95		1.66	
			240	35.72	64.87	29.15	3.89		1.75	
971	977	GAISSVL	60	36.89	65.42	28.54	3.27	0.14	1.43	0.20
			120	39.95	69.95	30.01	3.50		1.50	
			240	36.46	68.59	32.13	3.43		1.61	
971	979	GAISSVLND	60	26.22	49.36	23.14	3.46	0.39	1.62	0.03
			120	26.40	51.37	24.97	3.60		1.75	
			240	23.04	50.56	27.52	3.54		1.93	
975	979	SVLND	60	14.35	61.79	47.44	1.85	0.12	1.42	0.11
			120	9.04	63.94	54.89	1.92		1.65	
			240	12.13	67.79	55.65	2.03		1.67	
975	981	SVLNDIL	60	25.21	55.67	30.46	2.78	0.29	1.52	0.19
			120	29.11	62.46	33.35	3.12		1.67	
			240	36.68	66.95	30.27	3.35		1.51	
978	981	NDIL	60	41.41	42.08	0.66	0.84	0.09	0.01	0.02
			120	57.30	58.30	1.00	1.17		0.02	
			240	65.31	66.18	0.87	1.32		0.02	
978	990	NDILSRLDPPEAE	60	48.34	48.51	0.17	4.37	1.38	0.02	2.54
			120	51.99	57.88	5.90	5.21		0.53	
			240	55.78	62.84	7.06	5.66		0.64	
978	1001	NDILSRLDPPEAEVQIDRLITGRL	60	34.50	36.95	2.44	7.39	0.88	0.49	0.23
			120	42.36	45.49	3.13	9.10		0.63	
			240	49.20	52.05	2.85	10.41		0.57	
980	989	ILSRLDPPEA	60	41.65	46.06	4.42	2.76	0.45	0.27	0.01
			120	46.58	51.09	4.50	3.07		0.27	
			240	47.14	52.01	4.87	3.12		0.29	
980	990	ILSRLDPPEAE	60	41.89	46.53	4.63	3.26	0.38	0.32	0.06
			120	47.04	51.89	4.84	3.63		0.34	
			240	46.70	51.83	5.14	3.63		0.36	
980	992	ILSRLDPPEAEVQ	60	41.93	46.02	4.10	4.14	0.41	0.37	0.16
			120	48.28	52.42	4.13	4.72		0.37	
			240	48.83	53.46	4.63	4.81		0.42	
982	989	SRLDPPEA	60	46.12	50.62	4.51	2.03	0.18	0.18	0.03
			120	51.78	56.42	4.64	2.26		0.19	
			240	53.11	57.87	4.77	2.32		0.19	
982	990	SRLDPPEAE	60	44.73	49.97	5.25	2.50	0.15	0.26	0.04
			120	50.70	56.03	5.33	2.80		0.27	
			240	51.83	56.98	5.15	2.85		0.26	
982	992	SRLDPPEAEVQ	60	43.10	47.82	4.73	3.35	0.32	0.33	0.06
			120	50.12	55.09	4.97	3.86		0.35	
			240	52.26	57.18	4.93	4.00		0.35	
982	996	SRLDPPEAEVQIDRL	60	33.88	37.07	3.20	4.08	1.20	0.35	0.25
			120	40.16	43.43	3.27	4.78		0.36	
			240	43.04	46.78	3.74	5.15		0.41	
982	1001	SRLDPPEAEVQIDRLITGRL	60	27.77	30.83	3.06	4.93	0.51	0.49	0.17
			120	34.11	36.91	2.79	5.91		0.45	
			240	37.30	40.51	3.22	6.48		0.51	
990	996	EVQIDRL	60	37.59	41.59	4.00	2.08	0.26	0.20	0.07
			120	47.04	51.40	4.36	2.57		0.22	
			240	51.71	55.89	4.18	2.79		0.21	
990	1001	EVQIDRLITGRL	60	21.99	23.15	1.16	2.32	0.65	0.12	0.09
			120	28.45	29.72	1.27	2.97		0.13	
			240	34.79	36.40	1.61	3.64		0.16	
991	996	VQIDRL	60	30.43	36.99	6.56	1.48	0.16	0.26	0.18
			120	41.53	47.24	5.71	1.89		0.23	
			240	46.18	52.00	5.81	2.08		0.23	
991	998	VQIDRLIT	60	20.14	24.97	4.84	1.50	0.51	0.29	0.12
			120	27.89	33.50	5.61	2.01		0.34	
			240	27.86	34.04	6.18	2.04		0.37	
991	1001	VQIDRLITGRL	60	17.52	19.63	2.11	1.77	0.41	0.19	0.04
			120	24.44	26.78	2.34	2.41		0.21	
			240	30.25	32.56	2.31	2.93		0.21	
991	1006	VQIDRLITGRLQSLQT	60	14.02	15.96	1.93	2.23	0.72	0.27	0.13
			120	18.88	21.45	2.58	3.00		0.36	
			240	22.94	26.10	3.17	3.65		0.44	
992	1001	QIDRLITGRL	60	13.08	15.15	2.06	1.21	0.10	0.17	0.42
			120	17.04	21.47	4.44	1.72		0.36	
			240	26.57	27.53	0.96	2.20		0.08	
993	998	IDRLIT	60	19.65	23.33	3.68	0.93	0.36	0.15	0.13
			120	26.46	28.65	2.19	1.15		0.09	
			240	32.38	35.21	2.83	1.41		0.11	
993	1001	IDRLITGRL	60	11.69	13.75	2.06	0.96	0.17	0.14	0.04
			120	17.11	19.65	2.54	1.38		0.18	
			240	23.40	26.11	2.71	1.83		0.19	
994	1001	DRLITGRL	60	10.08	11.34	1.26	0.68	0.05	0.08	0.17
			120	16.83	18.37	1.54	1.10		0.09	
			240	21.60	24.05	2.46	1.44		0.15	
995	1001	RLITGRL	60	5.81	6.79	0.99	0.34	0.26	0.05	0.28
			120	9.33	12.20	2.86	0.61		0.14	
			240	16.16	17.72	1.56	0.89		0.08	
996	1000	LITGR	60	7.61	11.09	3.48	0.33	0.13	0.10	0.03
			120	13.23	16.79	3.56	0.50		0.11	
			240	19.12	22.80	3.68	0.68		0.11	
997	1000	ITGR	60	9.54	11.50	1.97	0.23	0.10	0.04	0.05
			120	16.49	19.09	2.60	0.38		0.05	
			240	25.67	27.50	1.84	0.55		0.04	
997	1001	ITGRL	60	7.61	11.09	3.48	0.33	0.13	0.10	0.03
			120	13.23	16.79	3.56	0.50		0.11	
			240	19.12	22.80	3.68	0.68		0.11	
997	1006	ITGRLQSLQT	60	6.86	10.29	3.44	0.82	0.15	0.28	0.04
			120	9.78	13.33	3.55	1.07		0.28	
			240	12.11	15.68	3.57	1.25		0.29	

To determine the binding sites (epitopes) of the CV804 antibody from the amino acid sequence of the SARS-CoV-2 spike protein (Genbank ACC No. QHD43416.1), mass spectrometry was performed using hydrogen/deuterium exchange epitope mapping. The hydrogen-deuterium exchange rates of each peptide were automatically calculated from the mass spectra obtained in the full-scan mode in the deuterium exchange experiment. Peptides with calculated hydrogen-deuterium exchange rates were filtered according to the criteria of a difference (ΔD%) in hydrogen-deuterium exchange rates between samples with and without the presence of hCV804-40 antibody in two or more adjacent peptides being 5% or more. The amino acid residues of the SARS-CoV-2 spike protein corresponding to the region containing peptides that met the criteria were identified as the epitope of the hCV804-40 antibody. The sequence coverage of the target protein was 98.5%, and a total of 737 peptides were identified in the samples with and without the presence of the hCV804-40 antibody. Among the identified 737 peptides, 36 peptides met the criteria of a difference (ΔD%) in hydrogen-deuterium exchange rates between samples with and without the presence of the hCV804-40 antibody being 5% or more. These 36 peptides were found to contain 42 amino acid residues (sequence number 49) corresponding to amino acid residues 960–1001 (sequence number 48) of the SARS-CoV-2 spike protein (Genbank ACC No. QHD43416.1). Therefore, amino acid residues of the SARS-CoV-2 spike protein to which the hCV804-40 antibody binds are located at positions 960–1001. The table data show the sequence numbers, amino acid sequences, positions of amino acid residues in the SARS-CoV-2 spike protein, and ΔD% for the above-mentioned 36 peptides. #1 represents the sample in the absence of hCV804-40 antibody, and #2 represents the sample in the presence of hCV804-40 antibody. Similar experiments were conducted for the CV804 antibody, and similar results were obtained, indicating that amino acid residues 960–1001 of the SARS-CoV-2 spike protein are also included in the epitope.

For the epitope identified from the HDX-MS analysis, we performed primary sequence alignment of the spike proteins from 27 related coronaviruses (Fig 4B). We identified amino acid residues that are common to all virus species as well as those that are characteristic to beta coronaviruses but differ from alpha coronaviruses, and mapped them onto the reported higher order structure of the CoV2 Spike 2-up form (PDB Code: 6BSV). As a result, many of the conserved amino acids in beta coronaviruses, which are included in the epitope region of CV804, were found to be located in exposed positions in the up form structure of the spike, strongly suggesting that the higher order structure composed of these residues is important for the binding of CV804 antibody (Fig 4C). In the alanine scan analysis of these mutants, the binding of CV804 antibody was abolished by mutations to alanine at residues L962, E988, Q992, L996, R1000, L1001, L1004, Y1007, and I1018, suggesting that these conserved residues are particularly important for the binding of CV804 to related coronaviruses (Fig 4D). In contrast, there were residues characteristic of alpha coronaviruses in the vicinity of these conserved residues, indicating a possible reason for the lack of cross-reactivity of CV804 with alpha coronaviruses. It should be noted that alanine mutations at these residues did not affect the reactivity of CV804, suggesting that the amino acid residues do not directly participate in the antigen-antibody interaction and are predicted to tolerate point mutations.

## Discussion

Antibody therapeutics against novel coronavirus infection should possess excellent specificity and efficacy. However, problems arise due to the emergence of virus variants resistant to nAbs, resulting in loss of efficacy, as well as difficulty in generating broadly reactive antibodies that can target diverse viruses. In this study, we successfully developed an anti-SARS-CoV-2 spike 2 antibody CV804 that exhibits therapeutic effects by utilizing antibody effector functions in the host, which is distinct from traditional virus-nAbs. Despite lacking virus-neutralizing activity, CV804 suppressed disease progression in a lethal infection mouse model to some extent. Furthermore, CV804 recognizes a unique epitope that is different from other antibody therapeutics targeting the critical site RBD in virus-infected individuals, supporting the host immune response and inducing therapeutic effects.

The location of the epitope core of CV804, identified by HDX-MS, encompasses approximately 40 amino acids, including a portion of the HR1 region in the S2 domain and a portion of the central helical domain in the primary structure of the spike. In the quaternary structure, this region is located at the S1 end of the trimeric S2 domain of the viral spike, surrounded by the S1 receptor-binding domain. The spike protein of SARS-CoV-2 adopts an equilibrium between the "down" and "up" conformations. Binding of the receptor-binding domain in the S1 domain to the ACE2 receptor on the surface of human cells, stabilizes the "up" conformation, in which S1 domain is cleaved from the S2 domain, consequently exposes S2 domain responsible for membrane fusion required for viral entry. The epitope structure of CV804 is located within the S2 region but is covered by the S1 domain in the initial state before receptor binding and positioned inside the trimeric spike structure surrounded by the S1 receptor-binding domain in the "down" conformation [29]. However, it becomes exposed when the spike protein adopts the "up" conformation [30, 31]. Due to the strong reactivity of CV804 towards cells expressing the spike protein, it is speculated that the exposed higher-order structure in the "up" conformation of the spike is present on the spike protein of infected cells. On the other hand, since CV804 does not exhibit *in vitro* infection-blocking activity (neutralizing activity), the binding of the antibody to this epitope region probably does not disturb against the membrane fusion required for virus infection. Structural data shown in Fig 4 indicate that several key residues involved in the antibody-antigen interaction are either completely or partially hidden within the pre-fusion S trimer. This suggests that structural changes occur during the transition from the pre-fusion state to the post-fusion state, leading to increased epitope exposure and enabling binding of CV804. The lack of neutralizing capability of CV804 could be due to limited epitope exposure. However, CV804 exhibits a high degree of cross-reactivity with various mutant strains and related coronaviruses, indicating that the epitope structure of CV804 is shared among many related coronaviruses.

The S2 region of coronaviruses is highly conserved and represents a promising target for a wide range of coronavirus antibodies and vaccines, compared to the S1 region [18, 31–33]. Several nAbs such as S2P6, CC40.8, IgG22, B6, 28D9, and CV3-25 have been isolated from convalescent patients, vaccinated individuals, and mice [13, 14, 34–36]. Consistent with the highly conserved S2 stem helix epitope among beta-coronaviruses, these nAbs typically demonstrate a broad neutralizing breadth against beta-coronaviruses, including SARS-CoV-1 and SARS-CoV-2 [13, 14]. In our study as well, CV804 exhibited extensive reactivity against beta-coronaviruses, similar to or surpassing the aforementioned antibodies. The highly conserved epitope recognized by CV804 leads to its extensive reactivity. Mutations within the epitope residues of CV804 in SARS-CoV-2 sequences were found to be less than 0.032%, and all relevant SARS-CoV-2 variants do not have residue mutations within this epitope. Notably, major natural variations within the CV804 epitope did not affect its binding activity. Some mutations can significantly reduce viral infectivity, thus potentially limiting the spread of the virus. We concluded that CV804, with its broad spectrum, represents an attractive means to address viral escape mutations and should be a valuable tool in tackling future outbreaks of novel coronaviruses.

Neutralizing antibodies fundamentally prevent viruses from entering cells, but are not capable of inhibiting viral replication within cells. Consequently, while antibodies that block infection possess prophylactic efficacy, they are not anticipated to exhibit potent therapeutic effects. In contrast, CV804 binds to a conserved region of the S2 domain of the spike protein expressed in infected cells, and consequently could eliminate virus-infected cells, through ADCC activity. Whereas CV804 has no inhibitory effect of infection in *in vitro* evaluation, it shows efficacy in *in vivo* evaluation. Due to attenuation by LALA mutation, this efficacy is attributed to effector functions such as ADCC and ADCP. The efficacy potential of CV804 as a monotherapy was insufficient compared to previous nAbs. We need to confirm whether drug efficacy is enhanced by ADCC activity-enhancing mutations, and also consider human immunity differences, such as F or V variants, for therapeutics. The findings on the epitope analysis of CV804 in this study could be helpful for future development of broad-spectrum anti-coronavirus antibody therapeutics and further research on vaccine development to efficiently induce useful antibody responses.

## Supporting information

S1 Table Numbers of euthanized, dead or surviving mice in the study for examining the lethality in SARS-CoV-2 infected mice. (DOCX)

S1 Fig Antibody binding activity to S protein of HCoV-OC43. ELISA analysis was conducted using recombinant protein to assess the binding of CV804 to HCoV-OC43, another human coronavirus.
(TIF)

S2 Fig Evaluation of hCV804-40 antibody, REGN10987, and LY-CoV1404 antibody binding to cells infected with SARS-CoV-2 variants. To further understand the characteristics of CV804, we conducted a study on its binding to cells expressing spike protein post-infection using various mutant strains, including the Omicron variant. Unlike S1 antibodies such as REGN10987 and LY-CoV1404, CV804 exhibited binding activity against multiple strains, including B.1.1.7, B.1.351, P.1, B.1.617.1, B.1.617.2, and B.1.1.529 (BA.1), as well as the Omicron subvariants (BA.2.75, BA.4.1, BA.4.6, BE.1, BA.5.2.1, BQ.1.1, XBB.1, XE, BF.7, BF.7.4.1). The hCV804-40 antibody demonstrated spot confirmation for all strains. However, LY-Cov1404 and REGN10987 did not show spot confirmation for three to four types of omicron strains.
(TIF)

S1 Raw data (ZIP)
