# Supplementary material for: Discovery of anti-SARS-CoV-2 S2 protein antibody CV804 with broad-spectrum reactivity with various beta coronaviruses and analysis of its pharmacological properties in vitro and in vivo
Source: PLoS One. 2024 Dec 2;19(12):e0300297. doi: 10.1371/journal.pone.0300297 (PMC11611099; doi:10.1371/journal.pone.0300297)
Supplement: S1 Table — (DOCX) [file pone.0300297.s001.docx]

| Infectious dose  (TCID_50_) | Group | Dose (mg/kg) | Euthanized^a^ / Died^b^ / Survived^c^ / Total per group | | | | | | | Survival rate (%) |
| --- | --- | --- | --- | --- | --- | --- | --- | --- | --- | --- |
| – | Non-infected | – | 0 | / | 0 | / | 5 | / | 5 | 100 |
| 1.0 × 10^5^ | Isotype control | 40 | 0 | / | 5 | / | 0 | / | 5 | 0 |
|  | CV804 | 40 | 1 | / | 0 | / | 4 | / | 5 | 80 |
|  | CV804 LALA | 40 | 5 | / | 0 | / | 0 | / | 5 | 0 |
|  | REGN10987 | 40 | 0 | / | 0 | / | 5 | / | 5 | 100 |

a, euthanized according to humane endpoints; b, died before reaching humane endpoints; c, survived for 6 days after virus inoculation.
