# Supplementary figures and images for: Discovery of anti-SARS-CoV-2 S2 protein antibody CV804 with broad-spectrum reactivity with various beta coronaviruses and analysis of its pharmacological properties in vitro and in vivo
Source: PLoS One. 2024 Dec 2;19(12):e0300297. doi: 10.1371/journal.pone.0300297 (PMC11611099; doi:10.1371/journal.pone.0300297)

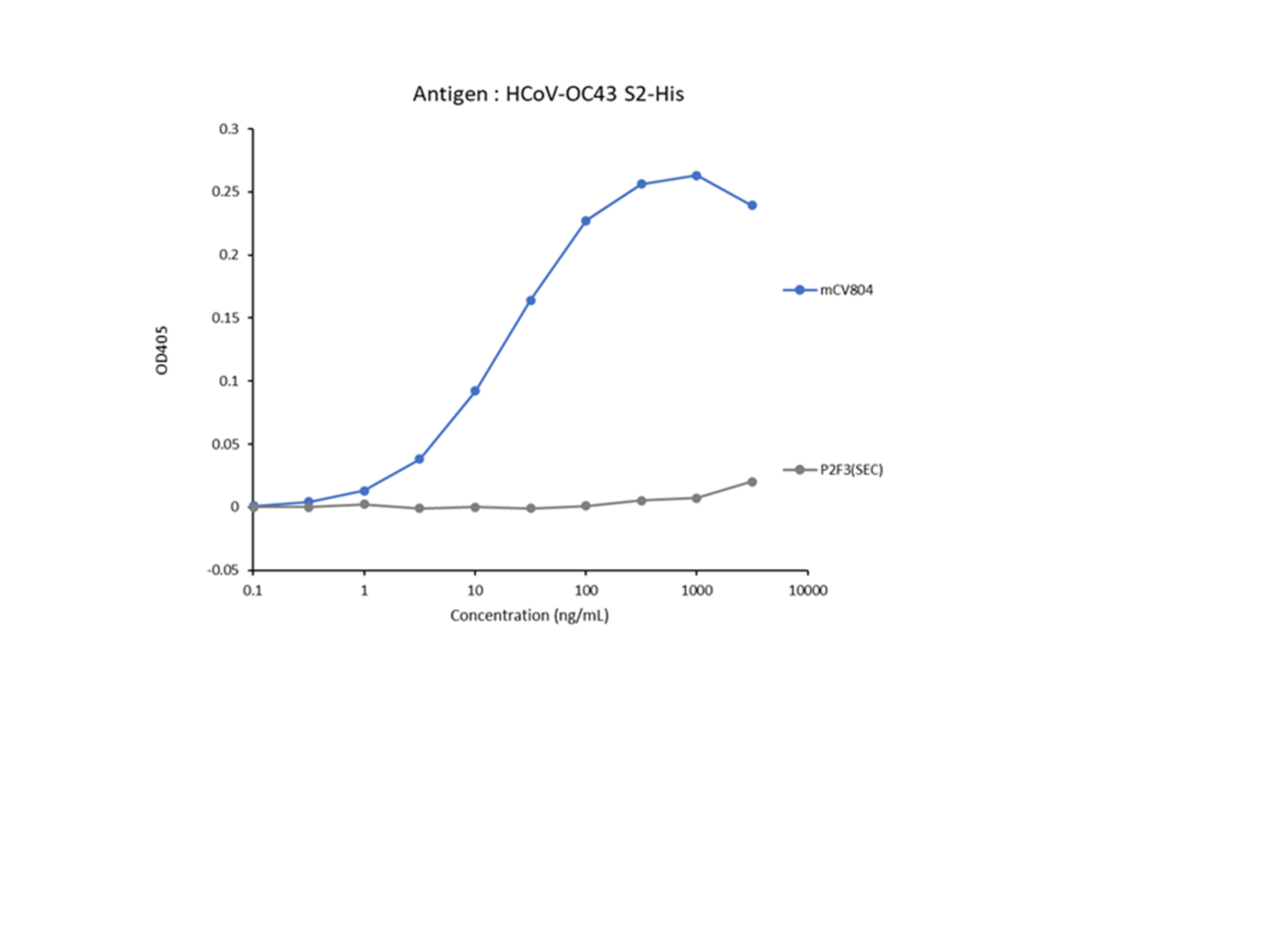

Supplement: S1 Fig — ELISA analysis was conducted using recombinant protein to assess the binding of CV804 to HCoV-OC43, another human coronavirus. (TIF) [file pone.0300297.s002.tif]

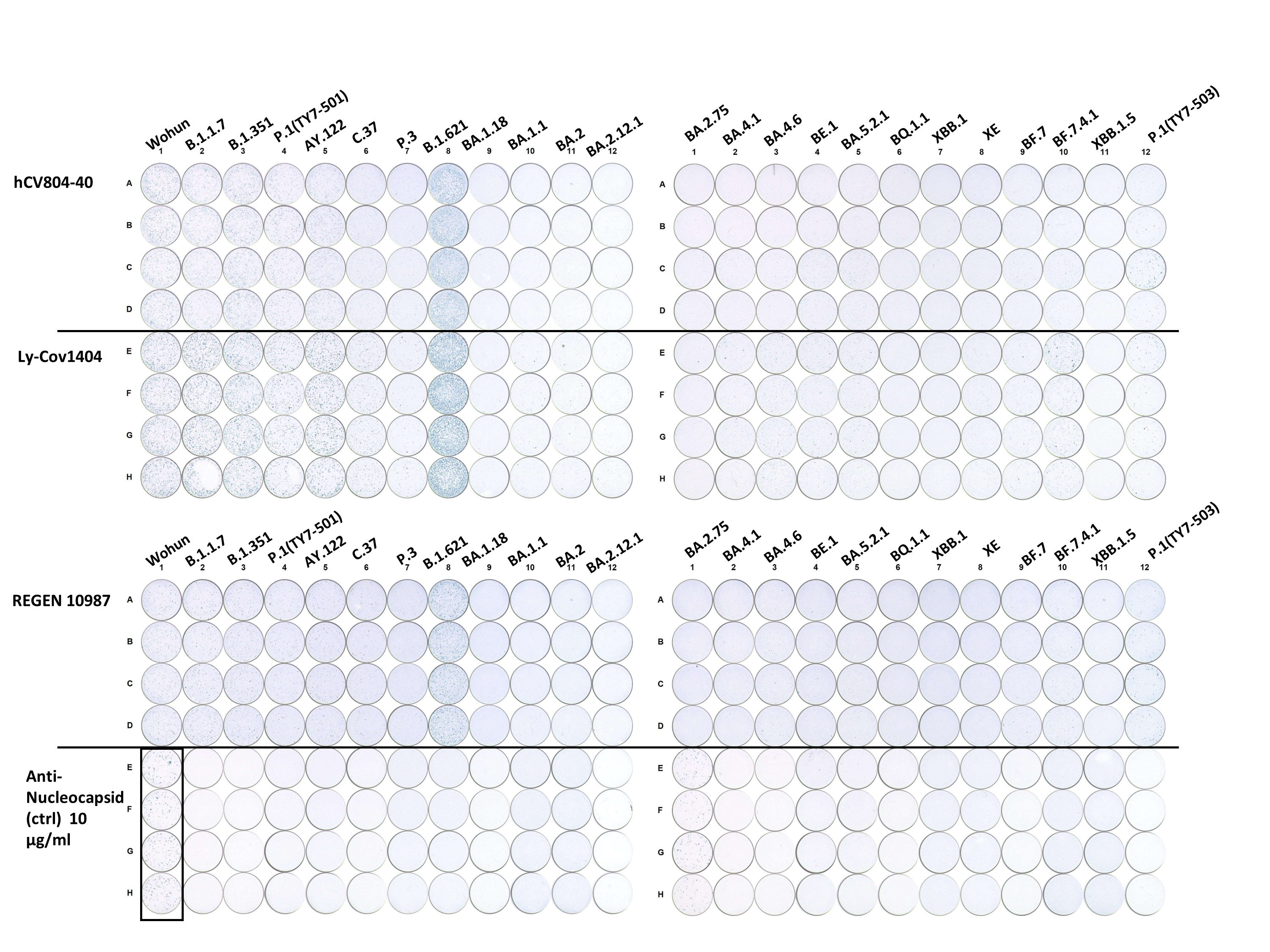

Supplement: S2 Fig — To further understand the characteristics of CV804, we conducted a study on its binding to cells expressing spike protein post-infection using various mutant strains, including the Omicron variant. Unlike S1 antibodies such as REGN10987 and LY-CoV1404, CV804 exhibited binding activity against multiple strains, including B.1.1.7, B.1.351, P.1, B.1.617.1, B.1.617.2, and B.1.1.529 (BA.1), as well as the Omicron subvariants (BA.2.75, BA.4.1, BA.4.6, BE.1, BA.5.2.1, BQ.1.1, XBB.1, XE, BF.7, BF.7.4.1). The hCV804-40 antibody demonstrated spot confirmation for all strains. However, LY-Cov1404 and REGN10987 did not show spot confirmation for three to four types of omicron strains. (TIF) [file pone.0300297.s003.tif]
